# Supplementary material for: Using Optical Quality Analysis System for predicting surgical parameters in age-related cataract patients
Source: PLoS One. 2020 Oct 12;15(10):e0240350. doi: 10.1371/journal.pone.0240350 (PMC7549767; doi:10.1371/journal.pone.0240350)
Supplement: Attachment — Submitted filename: R2_Reponse to reviewers.docx [file pone.0240350.s002.docx]

PONE-D-20-08844

Using Optical quality analysis system for cataract preoperative assessment & phacodynamics prediction in age-related cataract patients

PLOS ONE

Saint-Etienne, August 29^th^ 2020

Dear Dr. Grulkowski,

Please find attached a revised version R2 of our article "Using Optical Quality Analysis System for predicting surgical parameters in age-related cataract patients".

We updated all the points concerning Journal Requirements. The authors received no specific funding for this work.

We have carefully considered and responded to all the points addressed by the reviewers.

We greatly hope that this new version will meet the reviewers' expectations and comply with your editorial policy.

Yours sincerely,

Dr. Thibaud GARCIN, M.D., Ph.D., FEBO

t.garcin@univ-st-etienne.fr

PONE-D-20-08844R1

Using Optical Quality Analysis System for predicting surgical parameters in age-related cataract patients

PLOS ONE

Dear Dr. Garcin,

Thank you for submitting your manuscript to PLOS ONE. After careful consideration, we feel that it has merit but does not fully meet PLOS ONE’s publication criteria as it currently stands. Therefore, we invite you to submit a revised version of the manuscript that addresses the points raised during the review process.

Please submit your revised manuscript by Sep 13 2020 11:59PM. If you will need more time than this to complete your revisions, please reply to this message or contact the journal office at plosone@plos.org. When you're ready to submit your revision, log on to https://www.editorialmanager.com/pone/ and select the 'Submissions Needing Revision' folder to locate your manuscript file.

Please include the following items when submitting your revised manuscript:

A rebuttal letter that responds to each point raised by the academic editor and reviewer(s). You should upload this letter as a separate file labeled 'Response to Reviewers'.

A marked-up copy of your manuscript that highlights changes made to the original version. You should upload this as a separate file labeled 'Revised Manuscript with Track Changes'.

An unmarked version of your revised paper without tracked changes. You should upload this as a separate file labeled 'Manuscript'.

If you would like to make changes to your financial disclosure, please include your updated statement in your cover letter. Guidelines for resubmitting your figure files are available below the reviewer comments at the end of this letter.

If applicable, we recommend that you deposit your laboratory protocols in protocols.io to enhance the reproducibility of your results. Protocols.io assigns your protocol its own identifier (DOI) so that it can be cited independently in the future. For instructions see: http://journals.plos.org/plosone/s/submission-guidelines#loc-laboratory-protocols

We look forward to receiving your revised manuscript.

Kind regards,

Ireneusz Grulkowski, PhD

Academic Editor

PLOS ONE

Journal Requirements:

Additional Editor Comments (if provided):

Please, address additional comments of the reviewers.

[Note: HTML markup is below. Please do not edit.]

Reviewers' comments:

Reviewer's Responses to Questions

Comments to the Author

1. If the authors have adequately addressed your comments raised in a previous round of review and you feel that this manuscript is now acceptable for publication, you may indicate that here to bypass the “Comments to the Author” section, enter your conflict of interest statement in the “Confidential to Editor” section, and submit your "Accept" recommendation.

Reviewer #1: All comments have been addressed

Reviewer #2: All comments have been addressed

Reviewer #3: All comments have been addressed

2. Is the manuscript technically sound, and do the data support the conclusions?

The manuscript must describe a technically sound piece of scientific research with data that supports the conclusions. Experiments must have been conducted rigorously, with appropriate controls, replication, and sample sizes. The conclusions must be drawn appropriately based on the data presented.

Reviewer #1: Yes

Reviewer #2: Partly

Reviewer #3: Yes

3. Has the statistical analysis been performed appropriately and rigorously?

Reviewer #1: Yes

Reviewer #2: Yes

Reviewer #3: Yes

4. Have the authors made all data underlying the findings in their manuscript fully available?

The PLOS Data policy requires authors to make all data underlying the findings described in their manuscript fully available without restriction, with rare exception (please refer to the Data Availability Statement in the manuscript PDF file). The data should be provided as part of the manuscript or its supporting information, or deposited to a public repository. For example, in addition to summary statistics, the data points behind means, medians and variance measures should be available. If there are restrictions on publicly sharing data—e.g. participant privacy or use of data from a third party—those must be specified.

Reviewer #1: Yes

Reviewer #2: Yes

Reviewer #3: Yes

5. Is the manuscript presented in an intelligible fashion and written in standard English?

PLOS ONE does not copyedit accepted manuscripts, so the language in submitted articles must be clear, correct, and unambiguous. Any typographical or grammatical errors should be corrected at revision, so please note any specific errors here.

Reviewer #1: Yes

Reviewer #2: No

Reviewer #3: Yes

6. Review Comments to the Author

Please use the space provided to explain your answers to the questions above. You may also include additional comments for the author, including concerns about dual publication, research ethics, or publication ethics. (Please upload your review as an attachment if it exceeds 20,000 characters)

Reviewer #1: (No Response)

Reviewer #2: Dear Thibaud Garcin,

Thank you for submitting in PLOS ONE your research work described in the manuscript “Using Optical Quality Analysis System for predicting surgical parameters in age-related cataract patients”. The new version of the manuscript has improved considerable in clearly and comprehension terms. However, I would like to comment some small details that I have been able to observe in this review:

• First of all, I would like to suggest a deep revision of the manuscript in order to improve some expressions and correct grammar mistakes. Although, in general terms the text is comprehensible, there are some sections where there are incoherent expressions and not well-written. I do recommend to send the manuscript to an expert or native speaker to improve the writing quality. The manuscript has now been reviewed again, this time by a native English speaker.

Introduction

• I think that the definition of the Strehl ratio (SR) is not correct. The SR does not depend on the Modulate Transfer Function (MTF). The SR is the ration between the peak intensity from the Point Spread Function (PSF) of the aberrated eye and the peak intensity from the PSF of the non-aberrated eye. Please, correct lines 113 to 115. Thank you for this comment. We updated the definition.

Material and Methods

a) Patients and ethic statement

• The 2 last paragraph are not well located in this section and it makes confusion to the reader. In lines 132 – 135, it is described that the only inclusion criteria is to obtain good OSI measurements. However, it is not known any exclusion criteria and ophthalmic exam until next section. Similarly, it is observed in the last paragraph (lines 136 – 141), where some subjects were excluded because of poor cooperation and dense cataracts, without knowing any details from phacodynamics mode adjustment. I would recommend to move this section before the section “statistical analysis” to describe previously inclusion and exclusion methods. Thank you for this comment. We create an “inclusion & exclusion criteria” section just before statistical analysis section.

Results

a) Correlation Analysis: Main objective, subjective preoperative parameters and phacodynamics.

• In order to avoid misunderstanding, I do recommend strongly to indicate “LogMar scale” in the axis Best-Corrected Visual Acuity (BCVA) in figures 1, 5 and 6. The current values can confuse to the reader with decimal scale and the interpretation of the Spearman coefficient can be opposite. Thank you for this comment. We have updated figures 1, 5 and 6.

b) Correlation Analysis: Other OQAS parameters, subjective preoperative parameters and phacodynamics.

• Relations between Optical Scattering Index (OSI) with Modulate Transfer Function (MTF) in lines 273 – 274; and between Strehl ratio (SR) with MTF in lines 281 – 282, should not be included in the manuscript. These correlations were well developed and thoroughly analysed by the company in order to determine the performance of the device. Consequently, these data are not going to give any particular and novel information. Thank you for this comment. The data in question have been deleted.

Discussion

• I do not really agree with the idea that OQAS and LOCSIII measures the same physical phenomenon. Specially, because OQAS measures the quality of the whole eye, and a simple uveitis or a corneal scar can affect completely the measurement. I would be more specific in this sentence (lines 390 – 392) explaining that both methods will be measuring the same parameters if there are no more ocular pathologies. Thank you for this comment. The clarification has been made.

Reviewer #3: Thank you for your answers and comments. You have solved most of my concerns successfully. Regarding language, it has improved significantly and it makes the text much easier to follow now. On the other hand, I personally think that IQR=Q-Q3 provides an easier explanation for the data dispersion and allows the comparison with SD (line 187 and tables). Thank you for this comment.

Some specific comments:

- Line 90: Repetition of a fragment. Thank you for this comment. The repetition has been removed.

- Lines 210 – 211: The sentence needs revising for a better understanding. The sentence was revised.

- Lines 240 – 242: Lack of statistical significance for correlation between LOCSIII C and CDE as well as US is probably due to insufficient number of patients in this subgroup (LOCSIII C). Notice that rs is > 0,580. You should comment it here and explain it in discussion. As section results must be factual, we have now explained it in the discussion, per your suggestion.

- Figures 5 and 6: The top right and bottom left graphs seem to be wrong e.g. n = 21? Thank you for this comment. The titles of the top-right and bottom-left graphs were inverted. This mistake has now been corrected.

- I would be very grateful if you could send me the data rs and p for the different subgroups of LOCSIII components because note (lines 235 – 237) that they are not shown in Figure 1.

We found significant correlations between BCVA and LOCSIII total (rs = 0.561 ; p=0.008 ; Y = 6.258*X + 2.575) (Fig 1) as well as in subgroups of different LOCSIII components

N rs = 0.633 ; p=0.005 ; Y = 4.738*X + 2.678

NC rs = 0.621 ; p=0.006 ; Y = 2.294*X + 1.338

NO rs = 0.581 ; p=0.012 ; Y = 2.444*X + 1.340

except for LOCSIII C rs = 0.007 ; p=0.696 ; Y = -0.746*X + 2.284

7. PLOS authors have the option to publish the peer review history of their article (what does this mean?). If published, this will include your full peer review and any attached files.

If you choose “no”, your identity will remain anonymous but your review may still be made public.

Do you want your identity to be public for this peer review? For information about this choice, including consent withdrawal, please see our Privacy Policy.

Reviewer #1: No

Reviewer #2: No

Reviewer #3: No

[NOTE: If reviewer comments were submitted as an attachment file, they will be attached to this email and accessible via the submission site. Please log into your account, locate the manuscript record, and check for the action link "View Attachments". If this link does not appear, there are no attachment files.]

While revising your submission, please upload your figure files to the Preflight Analysis and Conversion Engine (PACE) digital diagnostic tool, https://pacev2.apexcovantage.com/. PACE helps ensure that figures meet PLOS requirements. To use PACE, you must first register as a user. Registration is free. Then, login and navigate to the UPLOAD tab, where you will find detailed instructions on how to use the tool. If you encounter any issues or have any questions when using PACE, please email PLOS at figures@plos.org. Please note that Supporting Information files do not need this step.
